# T Cell Receptor Vβ Staining Identifies the Malignant Clone in Adult T cell Leukemia and Reveals Killing of Leukemia Cells by Autologous CD8^+^ T cells

**DOI:** 10.1371/journal.ppat.1006030

**Published:** 2016-11-28

**Authors:** Aileen G. Rowan, Aviva Witkover, Anat Melamed, Yuetsu Tanaka, Lucy B. M. Cook, Paul Fields, Graham P. Taylor, Charles R. M. Bangham

**Affiliations:** 1 Section of Virology, Department of Medicine, Imperial College London, London, United Kingdom; 2 Department of Immunology, Graduate School of Medicine, University of the Ryukyus, Okinawa, Japan; 3 Guy’s and St Thomas’ Hospital, London, United Kingdom; Miller School of Medicine, UNITED STATES

## Abstract

There is growing evidence that CD8^+^ cytotoxic T lymphocyte (CTL) responses can contribute to long-term remission of many malignancies. The etiological agent of adult T-cell leukemia/lymphoma (ATL), human T lymphotropic virus type-1 (HTLV-1), contains highly immunogenic CTL epitopes, but ATL patients typically have low frequencies of cytokine-producing HTLV-1-specific CD8^+^ cells in the circulation. It remains unclear whether patients with ATL possess CTLs that can kill the malignant HTLV-1 infected clone. Here we used flow cytometric staining of TCRVβ and cell adhesion molecule-1 (CADM1) to identify monoclonal populations of HTLV-1-infected T cells in the peripheral blood of patients with ATL. Thus, we quantified the rate of CD8^+^-mediated killing of the putative malignant clone in ex vivo blood samples. We observed that CD8^+^ cells from ATL patients were unable to lyse autologous ATL clones when tested directly ex vivo. However, short in vitro culture restored the ability of CD8^+^ cells to kill ex vivo ATL clones in some donors. The capacity of CD8^+^ cells to lyse HTLV-1 infected cells which expressed the viral sense strand gene products was significantly enhanced after in vitro culture, and donors with an ATL clone that expressed the HTLV-1 Tax gene were most likely to make a detectable lytic CD8^+^ response to the ATL cells. We conclude that some patients with ATL possess functional tumour-specific CTLs which could be exploited to contribute to control of the disease.

## Author Summary

Human T lymphotropic virus-1 infects T cells, causing them to multiply. In some people, cellular replication is unchecked, resulting in an aggressive blood cancer called adult T-cell leukemia/lymphoma. The virus proteins are efficiently recognised as ‘foreign’ by the immune system in most infected individuals. People with cancer have weak immune responses to certain viral proteins, however it was not known whether the immune system can attack the malignant cells in this disease. In this paper, we developed a method which allows us to directly monitor malignant cells, and used it to test whether malignant and non-malignant infected cells are killed by immune cells from people with the cancer. We found that some people had immune cells which could kill the cancer cells. These observations are both new and important because they raise the possibility of boosting the immune response to malignant cells as a novel therapeutic strategy for this aggressive and hard-to-treat disease.

## Introduction

Adult T cell leukemia/lymphoma is a mature T cell malignancy caused by the retrovirus human T lymphotropic virus-1 (HTLV-1). Four clinical subtypes exist: acute, lymphoma, chronic and smouldering, which range from highly aggressive to indolent in their clinical course [1,2]. Advances in chemotherapy protocols have contributed only a modest increase in overall survival of aggressive subtypes, and few patients receive potentially curative allogeneic hematopoietic stem cell transplantation (HSCT)[3]. Antiviral drugs (zidovudine and interferon alpha, AZT/IFN)[4–7] and molecular targeted therapy (anti-CCR4, Mogamulizumab)[8–10] have shown promising results, especially in chronic ATL, but their efficacy in the lymphoma and acute subtypes is limited. There is an urgent need for new therapies and strategies to consolidate existing treatments.

HTLV-1 establishes persistent infection by integration of the provirus into the genomic DNA of T lymphocytes, and propagates in the host by both clonal proliferation and cell-to-cell transmission[11,12]. Expression of structural genes on the sense strand of the 9kb genome is induced by the viral transcriptional transactivator protein Tax, triggering production of viral particles, cellular activation and proliferation. The antisense strand encodes HTLV-1 b-zip protein (HBZ), which opposes many of the actions of Tax[13]. HTLV-1^+^ individuals carry thousands of long-lived infected CD4^+^ clones in their peripheral blood, each of which has arisen from a single infection event[12,14]. Malignant cells in ATL are HTLV-1-infected clones: in 91% of ATL cases a single dominant proviral integration site makes up over 35% of the proviral load[15], circulating alongside subdominant populations of polyclonal infected and uninfected T cells. Although the genomic integration site influences clonal proliferation and proviral gene expression[16], it does not appear to explain clonal dominance in most cases of ATL[15]. Spontaneous mutations in the T cell receptor (TCR)/NF-kB[17], CCR4[18], p53[19] and, Notch-1[20] signalling pathways are frequently observed in malignant clones.

Several lines of evidence indicate that the outcome of HTLV-1 infection is determined by the equilibrium set between proliferation of infected cells and the activity of abundant, chronically activated, HTLV-1-specific cytotoxic T lymphocytes [21,22]. Major histocompatibility complex (MHC) class 1 alleles HLA-A*0201 and C*08 are associated with a low proviral load[23] in southern Japan. Tax protein is highly immunodominant in the HTLV-1-specific CD8^+^ response, and *tax* is silenced or deleted in the dominant clone in over 50% of patients with ATL, implying the presence of strong CTL selection pressure. Paradoxically, ectopic expression of Tax can be oncogenic in vivo[24]. The region of the viral genome which encodes HBZ is highly conserved in ATL[25], suggesting HBZ also has a role in oncogenesis. The ability of an individual to present peptides from HBZ to CD8^+^ cells is associated with a low proviral load[26], however, HBZ evades immune detection by means of low-level protein expression and weak immunogenicity[26]. In addition, biological actions are exerted by untranslated HBZ mRNA[25,27].

ATL patients are commonly immunosuppressed, and frequently present with opportunistic infections. Previous studies on samples from ATL patients have reported that the frequency and diversity of HTLV-1-specific CD8^+^ T cells is significantly lower in ATL patients than in non-malignant HTLV-1 infection[28,29]. In addition to the silencing of Tax expression, several mechanisms by which ATL cells might escape CTL have been proposed. The malignant clone in 5%-6% of ATL patients carries mutations in *HLA-A* or *-B* genes, and the MHC class 1-encoding region in ATL is frequently subject to hypermethylation and copy-number variation[17]. ATL cells frequently express the regulatory T-cell-associated transcription factor FoxP3[30] and the coinhibitory ligand PD-L1[31], but it remains unclear whether primary ATL clones directly suppress CD8^+^ responses. Indeed, the susceptibility of primary ATL clones to CD8^+^-mediated lysis is not known, though rare occurrences of spontaneous disease remission[32], and successful allogeneic HSCT[33,34] have been reported to involve induction and maintenance of HTLV-1-specific CTLs[35].

Measuring the rate at which ATL clones are killed by CD8^+^ cells requires a reliable method to distinguish ATL clones from both non-malignant HTLV-1 infected cells and uninfected T cells. We recently published that CADM1 expression identifies 60–70% of infected cells in HTLV-1 carriers [36]. ATL patients have high frequencies of CADM1^+^[37],CCR4^+^[38], CD25^+^[1] and CD7^−^[39] cells in their peripheral blood. These cells often express FoxP3[40] and low levels of CD3 epsilon [41]. However, this combination of markers is also expressed by a subset of CD4^+^ T cells in uninfected donors [42] and asymptomatic HTLV-1 carriers (ACs), particularly those with a high proviral load [39,43], thus may not be used to directly identify the ATL clone.

Here, we used TCRVβ subunit staining, immunophenotyping and high-throughput sequencing to identify clonally expanded populations in a well-characterised cohort of ATL patients. We show that in some individuals with ATL, the malignant clone is susceptible to lysis by cultured autologous CD8^+^ cells. Autologous CD8^+^ cells from ATL patients preferentially killed targets that expressed the viral sense strand: both Tax^+^ATL clones and Tax^+^non-malignant cells were killed. In all donors, cells which did not express Tax escaped killing by CD8^+^ cells.

## Results

### Flow cytometric quantification of TCRVβ subunits reveals expanded clones of HTLV-1-infected cells in ATL patients

Peripheral blood mononuclear cells from ATL patients, asymptomatic HTLV-1 carriers and patients with HTLV-1 associated myelopathy/tropical spastic paraparesis (HAM/TSP) were stained with a panel of antibodies specific for 24 TCRVβ subunits (S1 and S2 Tables) and CADM1.

**Fig 1:**
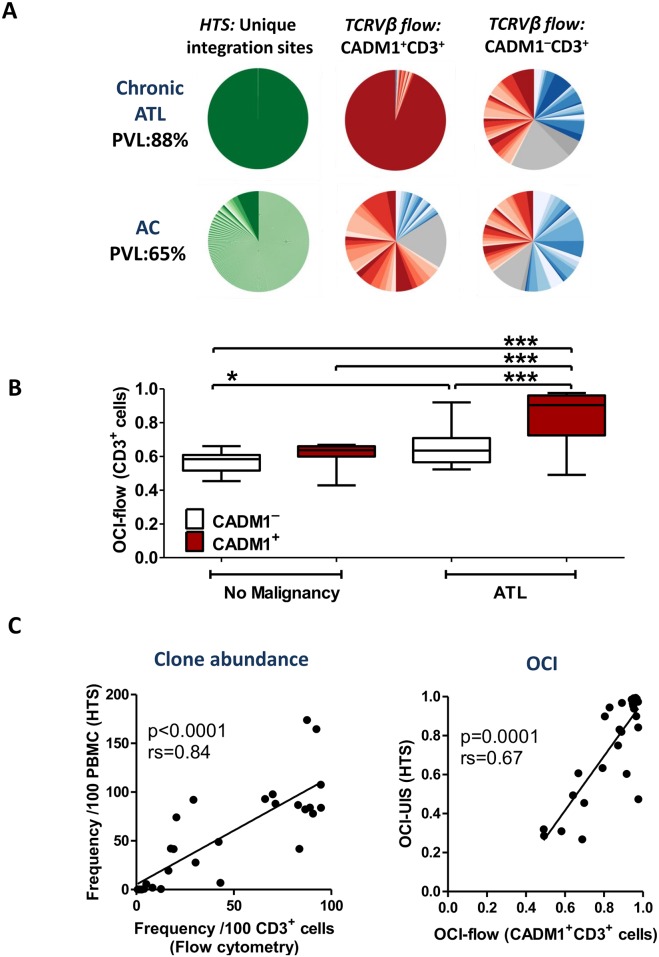
Flow cytometric staining of TCRVβ subunits reveals clonal expansions in ATL patients. Cryopreserved PBMCs from 52 individuals (28 ATL; 11 AC; 13 HAM) were thawed and stained with a viability stain followed by antibodies specific for 24 TCRVβ subunits, CD3, CD4, CD8, CADM1, CD7, CD127, CD25 and CCR4. Proviral genomic integration sites were mapped by LM-PCR and HTS. OCI was calculated using the Gini index, as previously described [12]. (A) Representative data from one individual with chronic ATL, and one high PVL AC. Pie charts show the relative frequency distribution of unique integration sites (green), and CD3^+^ cells (TCRVβ identified: CD4^+^, red; CD8^+^, blue; TCRVβ ‘off panel’: CD4^+^, light grey; CD8^+^, dark grey). (B) OCI-flow of CADM1^+^CD3^+^ cells versus OCI-flow of CADM1^−^CD3^+^ cells. Statistical analysis: Kruskal-Wallis test with Dunn post-test, 95% confidence interval (CI). * denotes p<0.05, *** denotes p<0.001. (C) Comparison of LM-PCR/HTS data (n = 28 ATL patients) and CADM1/TCRVβ flow cytometry. Statistical analysis: Spearman correlation.

The frequency distributions of TCRVβ subunits in CADM1^+^ (which typically carry one proviral copy per cell[36]) and CADM1^−^ (low proviral load) T cells (both CD4^+^ and CD8^+^) were ascertained by dividing live CD3^+^ cells into 50 possible groups on the basis of TCRVβ staining (see Materials and Methods). Linker-mediated PCR (LM-PCR) followed by high-throughput sequencing (HTS) were performed to corroborate the observed frequency distributions (Fig 1A).

We used an oligoclonality index (OCI, Gini index)[12] to compare the frequency distribution of TCRVβ subunits (OCI-flow) with the frequency distribution of unique proviral integration sites (UIS, OCI-UIS). The frequency distribution of TCRVβ subunits in ACs and patients with HAM/TSP resembled that in healthy donors [44] (S1A and S2 Figs), with no significant difference in the OCI-flow of infected CADM1^+^CD3^+^ cells and the OCI-flow of predominantly uninfected CADM1^−^CD3^+^ cells (Fig 1B). By contrast, the OCI-flow of CADM1^+^CD3^+^ cells in ATL patients was significantly higher than that of CADM1^−^CD3^+^ cells from the same donor, and CADM1^+^/^−^CD3^+^ cells from donors without malignancy (Fig 1B). These results indicate that an OCI-flow>0.7 is associated with ATL (see below). In ATL patients, the OCI-flow for CADM1^+^CD3^+^ T cells measured by flow cytometry was significantly correlated with the OCI-UIS measured by HTS (Fig 1C). In addition, the absolute frequency of the most abundant UIS detected by HTS was significantly correlated with the frequency of the most abundant population of T cells which shared a single Vβ subunit (Fig 1C). We therefore refer henceforth to the dominant TCRVβ-expressing population of CD4^+^ cells, in individuals with an OCI-flow (CADM1^+^CD3^+^) > 0.7, as the ‘ATL clone’.

We detected putatively malignant expansions in patients with chronic (n = 12) or acute (n = 6) leukemia (Fig 1B, S1B and S2 Figs); in 16 cases by direct identification of the TCRVβ and two cases in which the TCRVβ subunit was not represented in the TCRVβ antibody panel. Each case had a population of T cells which shared a Vβ subunit comprising >35% of CADM1^+^ cells[15], and an OCI-flow of CADM1^+^CD3^+^ cells > 0.7. There was no evidence of preferential transformation of cells expressing particular TCRVβ subunits (S1 Table). Two out of five lymphoma patients also had CADM1^+^CD3^+^ PBMC with an OCI-flow > 0.7. Patients with leukemic type ATL who had an OCI-flow (CADM1^+^CD3^+^) <0.7 were in remission, and did not have a dominant proviral integration site (>35% of the PVL) detectable by HTS.

### Comparison with other ATL cell markers

**Fig 2:**
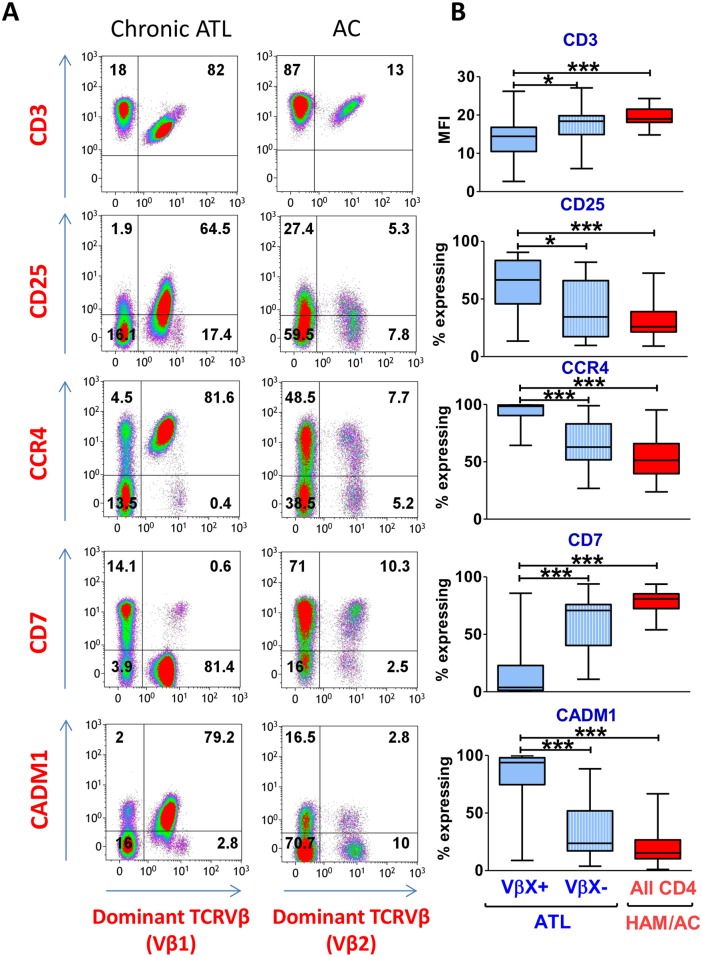
Expression of candidate ATL cell surface markers by the dominant TCRVβ-expressing population. Staining was performed as described in Fig 1. (A) Representative flow cytometry plots of total live CD3^+^CD4^+^ cells from an ATL patient (LHN) and an AC (HHD). Plots display the most frequently expressed TCRVβ subunit in the respective donor. (B) Expanded clones are CCR4^+^CD7^−^CADM1^+^. Live CD3^+^CD4^+^ T cells from ATL patients (n = 21) with an OCI-flow (CADM1^+^CD3^+^) >0.7 were gated on the basis of expression of the dominant TCRVβ (designated TCRVβX^+^ or TCRVβX^**–**^). Total live CD4^+^ T cells from PBMC of n = 24 individuals without malignancy (patients with HAM or ACs) were included as controls. Whiskers represent maximum and minimum values. Statistical analysis: Kruskal-Wallis test with Dunn post-test, 95% confidence interval (CI). * denotes p<0.05, ** denotes p<0.01, *** denotes p<0.001.

Direct flow-cytometric identification of clonal HTLV-1-infected populations in ATL permitted detailed assessment of the sensitivity and specificity of other established immunophenotypic markers of ATL. Using multicolour flow cytometry we evaluated co-expression of phenotypic ATL markers (CD3, CD25, CD7, CCR4 and CADM1) on cells which carried the respective dominant TCRVβ (designated TCRVβX^+^) with those which did not (TCRVβX^−^). As described in the literature, CD3 epsilon was significantly downregulated on expanded clones in ATL (Fig 2A and 2B), compared with cells from the same individual which expressed other TCRVβ subunits. CCR4 was expressed by a median of 98% cells within the malignant clone; CADM1 by 93%; and CD7 was downregulated on 96% (Fig 2B). CD25 had the poorest sensitivity of all the markers: a median of 66% of malignant cells were CD25^+^.

We tested the ability of CD7 and CD25 to discriminate between malignant and non-malignant infected cells by comparing the frequency of expression on CCR4^+^CADM1^+^CD4^+^cells from individuals with and without malignancy. Although expression of CD7 was significantly downregulated on infected CCR4^+^CADM1^+^ cells versus other CD4^+^ T cells in all HTLV-1-infected subjects, CD7 expression was lowest on CCR4^+^CADM1^+^ cells from ATL patients (S3 Fig). In contrast, the frequency of CD25 expression on CCR4^+^CADM1^+^ cells did not differ between the three disease states: ATL, HAM/TSP and AC (S3 Fig). After TCRVβ positivity, CD7 downregulation was the most specific marker of ATL clones. Thus, the combination of markers of clonal expansion (TCRVβ^+^ and CD7^low^) and infection (CADM1^+^) allows sensitive detection and accurate quantification of expanded ATL clones.

### OCI-flow(CADM1^+^CD3^+^) > 0.7 indicates a high probability of clinically evident ATL

**Fig 3:**
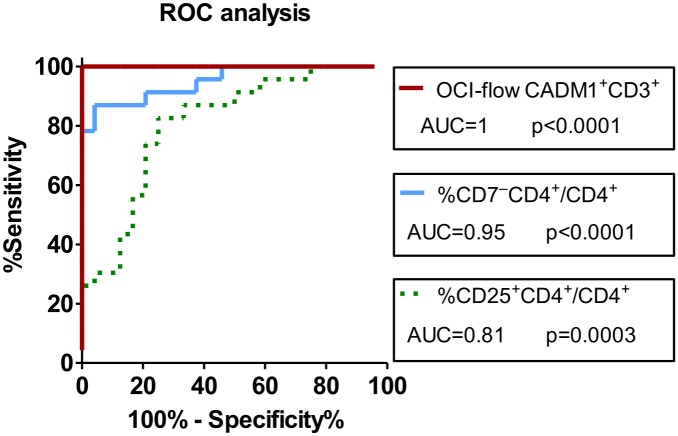
OCI-flow of CADM1^+^CD3^+^ cells is an excellent diagnostic test for monoclonal integration. Receiver operator curves illustrating the specificity and sensitivity by which the OCI-flow of CADM1^+^CD3^+^ cells, the frequency of CD7^−^CD4^+^ or the frequency of CD25^+^CD4^+^ cells discriminate individuals with clinically evident ATL (n = 23) from individuals with non-malignant HTLV-1 infection (n = 24). Individuals previously diagnosed with ATL which were in clinical remission were excluded from this analysis.

Within our cohort of 24 age-matched HTLV-1-infected individuals without malignancy (in whom the PVL ranged from undetectable to 79 copies per 100 PBMC, S1 Table), the OCI-flow of CADM1^+^CD3^+^ cells did not exceed 0.7. We plotted receiver operator curves (ROC) to evaluate the sensitivity and specificity by which the OCI-flow of CADM1^+^CD3^+^ cells could identify individuals with clinically evident ATL (Fig 3), compared with the common diagnostic investigations: enumerating CD7^−^CD4^+^ cells and CD25^+^CD4^+^ cells. Five ATL patients within the original cohort who were in clinical remission were excluded from this analysis on the basis of clinical observations (not on the basis of oligoclonality). Area under the curve (AUC) analysis rated the diagnostic power of the OCI-flow (CADM1^+^CD3^+^)and CD7^−^CD4^+^ frequency as ‘excellent’ (AUC 0.9–1), and CD25^+^CD4^+^ frequency as ‘good’ (AUC 0.8–0.9), and both tests had significantly higher diagnostic power than the frequency of CD25^+^CD4^+^ T cells (Fig 3, p = 0.001, OCI-flow (CADM1^+^CD3^+^) vs. CD25; p = 0.03, CD7 vs. CD25, one-tailed test [45]).

### ATL clones express MHC class 1 and high levels of CADM1

**Fig 4:**
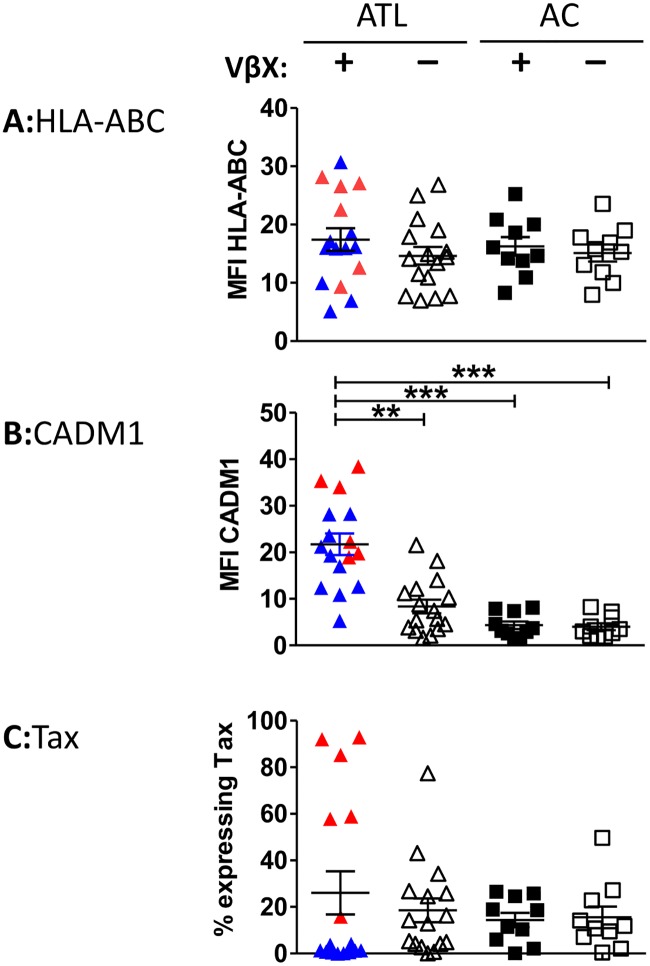
Expression HLA-ABC, CADM1 and Tax by CADM1^+^CD4^+^ T cells. CD8^+^ cells were depleted from PBMCs of 15 ATL patients with a dominant ATL clone detectable by TCRVβ staining, and 10 ACs. Cells were cultured for 18h, after which they were surface stained with a viability stain followed by antibodies specific for the most frequently utilised TCRVβ (VβX), CD3, CD4, CD8, CADM1, PD-L1, FoxP3 and HLA-ABC (S2 table; panels 3, 4, 6 and 7). Cells were then permeabilised and stained with antibodies specific for Tax and FoxP3 and analysed by flow cytometry. Cells from ATL patients and ACs were gated on live CD3^+^CD4^+^CADM1^+^ cells which were positive or negative for the dominant TCRVβX as indicated. Intensity of expression of (A) MHC class 1 and (B) CADM1. (C) Frequency of Tax expression by ATL clones. Tax^high^ ATL clones are plotted in red, and Tax^low^ ATL clones are plotted in blue. Statistical analysis: Kruskal-Wallis test with Dunn post-test, 95% CI. * denotes p<0.05, ** denotes p<0.01, *** denotes p<0.001.

In 16 individuals with a known dominant TCRVβ, all T cells (including ATL clones) expressed MHC class 1 at a similar intensity (Fig 4A and S4 Fig). CADM1 expression was significantly higher on ATL clones than on non-malignant infected cells within the same individual, or CADM1^+^ cells from ACs (Fig 4B and S4 Fig). In polyclonal infected populations (CD4^+^CADM1^+^ cells in ACs, CADM1^+^VβX^−^cells in ATL patients), a median of 11–15% of CADM1^+^ cells expressed Tax after overnight culture ([36]; Fig 4C and S4 Fig). By contrast, ATL clones fell into two distinct groups: those in which <5% of cells expressed Tax (Tax^low^ATL) and those in which >5% expressed Tax (Tax^high^ATL). FoxP3 and PD-L1 were highly expressed in some cases, by both Tax^high^ and Tax^low^ ATL clones (S5 Fig).

### Patient-derived CD8^+^ cells do not efficiently lyse ATL clones ex vivo

**Fig 5:**
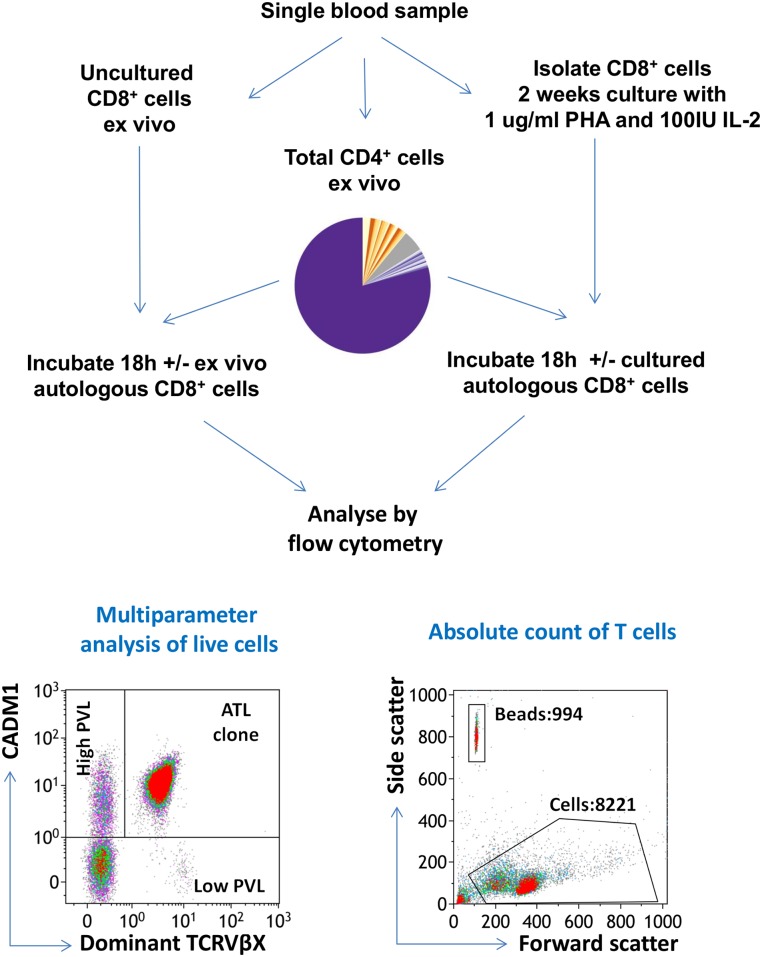
Experimental design of cell survival assay. PBMCs from each of 9 ATL patients with a dominant ATL clone detectable by TCRVβ staining were depleted of CD8^+^ T cells. The CD8^−^ PBMCs (CADM1^+^CD4^+^, purple; CADM1^−^CD4^+^, yellow) were cultured overnight either alone or in the presence of autologous CD8^+^ cells at a range of ratios, after which cells were stained with a viability stain and antibodies specific for CD3, CD4, CD8, CADM1 and the TCRVβ subunit which was most frequently used in that individual (‘TCRVβX’). Cells were then permeabilised, stained intracellularly with anti-Tax antibody, and analysed by flow cytometry. Absolute cell counts of CD3^+^, CD4^+^ and CD8^+^ cells were performed in parallel.

We tested the ability of autologous CD8^+^ cells to kill malignant clones using an ex vivo cell survival assay [46]. In order to mimic in vivo CD8^+^ cell:target cell frequencies, we incubated CD4^+^ PBMCs from ATL patients for 18h with a range of ratios of autologous CD8^+^ T cells and quantified the absolute number of surviving cells in the following populations: CADM1^−^CD4^+^ cells (which have a low proviral load [36]), malignant HTLV-1 infected CADM1^+^VβX^+^CD4^+^ cells, and non-malignant CADM1^+^VβX^−^CD4^+^ cells (Fig 5), which typically carry a single proviral copy per cell. This strategy permitted us to estimate the efficacy by which each subset was targeted by CD8^+^ cells in vivo, in the presence of other potential CTL targets. Because previous reports indicated that ex vivo CD8^+^ cells from ATL patients had negligible lytic function [28], purified CD8^+^ cells were expanded in culture for 2 weeks, both to increase the effector: target ratio, and to allow potential reactivation of lytic function.

**Fig 6:**
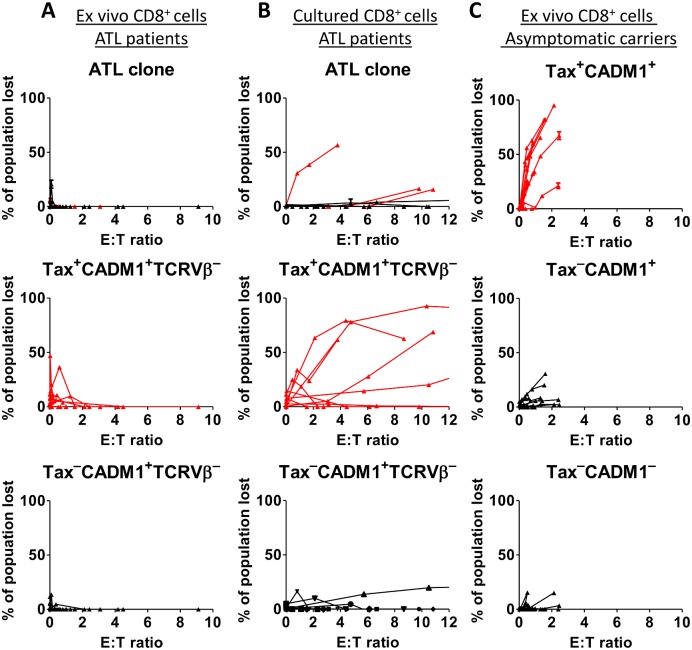
Cultured CD8^+^ cells can kill autologous malignant cells in some donors. CD8 depleted PBMCs from ATL patients (n = 9) with a known dominant TCRVβ were incubated in the presence of ex vivo (A) or cultured (B) autologous CD8^+^ cells at the indicated E:T ratios. After 18h, the absolute number of viable ATL cells (live CD3^+^CD4^+^CADM1^+^TCRVβ^+^) was quantified by flow cytometry and used to calculate the proportion of the ATL clone which had been specifically killed in the presence of CD8^+^ cells. The proportion of Tax^+^ and Tax^−^CADM1^+^ TCRVβ^−^ cells which were killed was calculated in the same manner. (C) Ex vivo CD8^+^ cell killing of Tax^+^CD3^+^CD4^+^CADM1^+^ cells, Tax^−^CD3^+^CD4^+^CADM1^+^ cells and CD3^+^CD4^+^CADM1^−^ cells in ACs (n = 10). Subsets of cells which expressed Tax (in both ATL and ACs) are plotted in red.

At the effector: target ratios tested, no significant lysis of the ATL clone by autologous ex vivo CD8^+^ T cells was detected (Fig 6A). When compared with ACs, CD8^+^ from ATL patients also had a markedly reduced ability to lyse non-malignant Tax-expressing CADM1^+^CD4^+^ cells (Fig 6). Tax^−^CADM1^+^ and CADM1^−^ CD4^+^ cells were not killed by ex-vivo CD8^+^ cells in either cohort. By contrast, after expansion in vitro, cultured CD8^+^ cells from 3 of 9 donors with ATL killed a proportion of their respective ATL clone (Fig 6B). Addition of 20nM concanamycin A blocked killing of ATL cells, indicating that the observed effect is perforin-dependent (S6 Fig), as previously reported [47].

### Efficiency of CTL selection: preferential lysis of Tax^high^ clones

**Fig 7:**
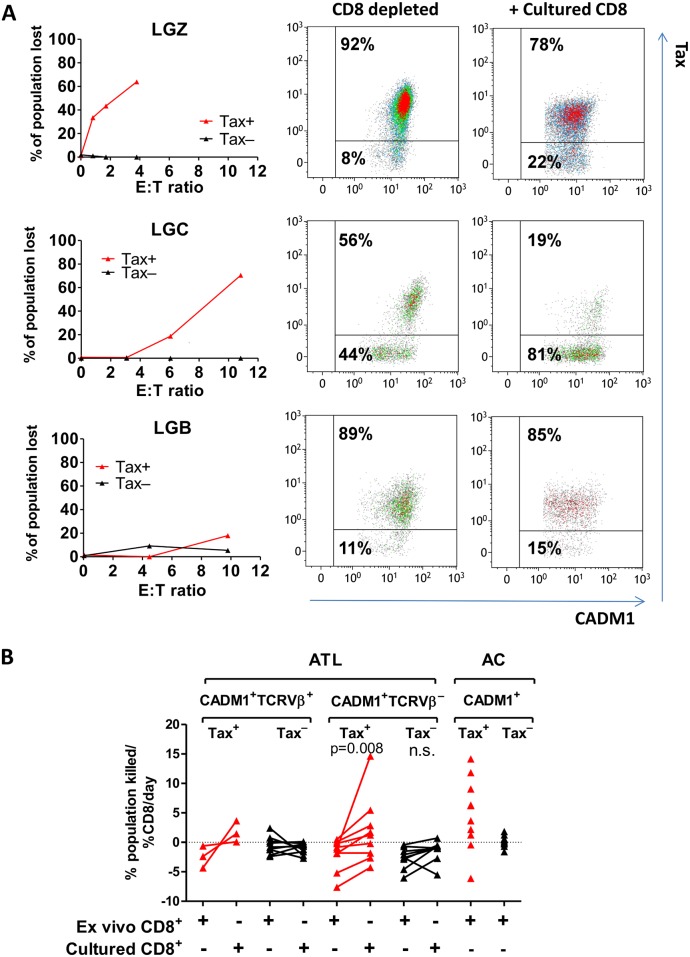
Tax-expressing cells are preferentially killed by cultured autologous CD8^+^ cells. (A) Selective loss of live Tax-expressing ATL cells after incubation with cultured CD8^+^ cells. Extended analysis of data from Fig 6. ATL clones from three Tax^high^ ATL patients (LGZ, LGC and LGB) were gated on the basis of Tax expression as shown. For each donor, graphs show the percentage of Tax^+^ or Tax^−^cells which were killed in the presence of cultured autologous CD8^+^ cells. Flow plots show Tax and CADM1 expression by live CD3^+^CD4^+^CADM1^+^TCRVβX^+^ cells from each individual after culture alone or in the presence of CD8^+^ cells at the highest E:T ratio tested. (B) Comparison of the rate at which ex vivo and cultured CD8^+^ cells supress survival of the populations indicated in ATL patients (n = 9) and ACs (n = 10). Subsets of cells which expressed Tax (in both ATL and ACs) are plotted in red. Data from Fig 6 was analysed by nonlinear regression to estimate the % change observed in each population with each 1% increase in CD8^+^ cells present in the co-culture. A negative rate indicates that number of viable target cells recovered from the co-culture was greater in the presence of CD8^+^ cells versus in the absence of CD8^+^ cells. Statistical analysis (CADM1^+^TCRVβ^−^ groups only): Wilcoxon matched pairs test, two tailed, 95% confidence interval.

We observed that the ATL clone was not completely eliminated at any CD4^+^:CD8^+^ ratio, even supraphysiological ratios (Fig 6). All donors (3/3) whose CD8^+^ cells regained the ability to lyse the malignant cells had an ATL clone which strongly expressed the proviral sense strand genes, as detected by intracellular expression of Tax protein (Tax^high^) (Fig 7A). In contrast, the malignant clones of all other donors in the cohort (6/6) were Tax^low^. Within the ATL clone, we observed a strong preferential lysis of Tax-expressing malignant cells; only one donor lysed Tax-negative malignant cells (Fig 7A). Between 20–60% of malignant Tax expressing cells were cleared in each donor (Fig 7A). To quantify the preferential CD8^+^ targeting of cells that express the viral plus strand, we calculated for each donor the rate at which Tax-expressing and non-expressing cells were killed after in vitro culture (Fig 7B). Cultured CD8^+^ cells killed Tax-expressing ATL clones at a higher rate than ex vivo CD8^+^ cells in 3 of 3 cases. In addition, cultured CD8^+^ cells from patients with Tax^low^ clones also had enhanced ability to kill non-malignant HTLV-1 infected cells which expressed the viral plus strand (Fig 7B).

## Discussion

An array of novel anti-cancer immune therapies are currently in clinical trials, which potentiate existing immune responses, and induce tumour-specific immunity by vaccination, or infusion of engineered tumour-specific T cells. Might these approaches be effective in ATL?

We demonstrate that abnormal clonal expansions of HTLV-1-infected T cells are readily detectable in individuals with ATL by TCRVβ flow cytometry, which is faster, cheaper and less labour-intensive than the current gold-standard technique of high-throughput sequencing of proviral integration sites. In this cohort, an oligoclonality index of > 0.7 within CADM1^+^CD3^+^cells reliably identified individuals who had a dominant ATL clone as validated by high-throughput sequencing. Whilst CD25 expression is frequently high in ATL, we show that in most individuals, ~40% of cells in the ATL clone are CD25 negative. Over 94% of ATL clones were CCR4^+^CADM1^+^CD7^−^; the exceptions were one CD7^dim^ ATL clone and one CADM1^−^ clone. Analysis of TCRVβ expression within the heavily infected CADM1^+^CD3^+^ population allows direct flow cytometric analysis of the clonal structure of HTLV-1 infected cells, and can distinguish ATL patients from age-matched HTLV-1 carriers with high specificity and sensitivity. Whilst we did not have sufficient cases to independently test the diagnostic power of OCI-flow of CADM1^+^CD3^+^ cells in an unrelated cohort of cases and controls, our observations indicate that this measure could be useful in the diagnosis of ATL: particularly in detecting the presence of monoclonal/oligoclonal populations of HTLV-1 infected cells.

We exploited this technique to measure the rate at which ATL clones are lysed by ex vivo, autologous CTLs. Ex vivo CD8^+^ cells were unable to kill autologous malignant ATL cells, even at supraphysiological E:T ratios. In addition, CD8^+^ mediated killing of non-malignant cells which express viral proteins was less efficient in ATL patients than in asymptomatic carriers. In certain patients, in vitro culture of CD8^+^ cells revealed a population of CD8^+^ cells which could kill the ATL clone. This ability was associated with expression of the viral plus-strand genes by the ATL clone: Tax^high^ATL clones and Tax expressing non-malignant infected cells were preferentially targeted by cultured autologous CD8^+^ cells.

In most subjects in the present study, Tax was expressed on <5% of cells within the ATL clone after overnight in vitro culture, and Tax-negative cells consistently escaped CD8^+^-mediated killing. We observed no defect in MHC class 1 expression by ATL clones, and the level of expression of CADM1 by ATL clones was significantly higher than that in non-malignant HTLV-1-infected cells. CADM1 expression on the target cell enhances its susceptibility to CTL killing [36,48]; thus CADM1 could contribute to CD8^+^ lysis of ATL clones. The level of expression of CADM1 did not significantly differ between Tax^high^ and Tax^low^ ATL clones: so while CADM1 is likely to facilitate killing of ATL clones which present epitopes which are recognised by CD8^+^ cells, CADM1 expression alone does not appear to expose the ATL clone to lysis by CD8^+^ cells. Likewise, the expression levels of PD-L1 and FoxP3 did not differ between Tax^high^ and Tax^low^ clones in our cohort, so we could not make any inferences on the role of FoxP3/PD-L1 in the escape of ATL cells in this study.

ATL clones have the potential to present a range of non-self antigens to CTL: for example, the consistently expressed HTLV-1 antigen HBZ[49], or neoepitopes generated by the frequent somatic mutations observed in ATL: a recent study detected 6404 somatic mutations in 81 ATL cases by exome sequencing [17]. Apart from the frequent loss of expression of the dominant CTL target antigen Tax by ATL clones, there was no evidence that these clones evaded the immune response by downregulation of MHC class 1; nevertheless, the bulk of ATL cells escaped CTL lysis in most individuals. We conclude that the CTL response to antigens presented by ATL clones is insufficient or suppressed in established disease.

Although the CD8^+^ response in patients with ATL appears insufficient to maintain control of ATL cell expansion in vivo, the capacity of autologous CD8^+^ cells to lyse the malignant clone that we report here indicates an opportunity for therapeutic intervention by boosting the CD8^+^ response, particularly in patients where the ATL clone expressed the viral plus strand. Immunisation strategies have focused on Tax, and more recently HBZ. Because Tax expression is intermittent or low in vivo, and frequently deleted in ATL clones, and HBZ is only weakly immunogenic [50], other HTLV-1 antigens or neoantigens may be more effective CD8^+^ epitopes. Whilst we observed CD8^+^ killing of Tax expressing cells within ATL clones, boosting Tax-specific CD8^+^ responses alone is likely to strongly select for deletion of Tax in malignant clones: clearly a CTL response which targets multiple antigens would reduce the likelihood of immune escape. While certain somatic mutations are observed in a high proportion of primary ATL clones[17], the combination of neoepitopes and the individual’s HLA type is unique for most individuals with ATL. Immunisation with epitopes from autologous ATL clones could elicit a broader cellular immune response to the malignancy in comparison with immunisation with HTLV-1 epitopes alone. Finally, the ability to maintain long-term populations of effector cells will be a critical factor determining the efficacy of the ATL-specific CD8^+^ response.

## Materials and Methods

### Ethics statement

Donors attended the National Centre for Human Retrovirology (Imperial College Healthcare NHS Trust, St Mary's Hospital, London). Written informed consent was obtained and research was conducted under the governance of the Communicable Diseases Research Group Tissue Bank, approved by the UK National Research Ethics Service (09/H0606/106, 15/SC/0089).

### Clinical samples

All ATL subtypes were included (S1 Table). PBMC were isolated from whole blood by density-gradient centrifugation using histopaque-1077 (Sigma-Aldrich, Poole) from EDTA-anticoagulated blood. Isolated PBMCs were washed twice in PBS then cryopreserved in FCS (Life technologies, Paisley) with 10% dimethysulfoxide (Sigma-Aldrich).

### Proviral load estimation and mapping of proviral integration sites

Genomic DNA was extracted using a DNeasy kit (Qiagen, Manchester), according to the manufacturer’s instructions, and proviral load was estimated as described in Manivannan et al, 2016 [36]. Genomic DNA (20 ng, 6.7 ng or 2.2 ng in 4 μl H_2_O) was subjected to thermal cycling in the presence of FastSYBR (Life Technologies) master mix and the following primer pairs: SK43/SK44- 5'CGGATACCCAGTCTACGTGT3' /5'GAGCCGATAACGCGTCCATCG3' (*tax* gene) or GAPDHF/GAPDHR- 5’AACAGCGACACCCATCCTC3’/5’ CATACCAGGAAATGAGCTTGACAA3’ (*gapdh* gene). DNA amplification was monitored in real time with a QuantStudio7 thermal cycler (Life technologies). DNA from a naturally-infected primary T cell clone which contained a single-copy of *tax* and two copies of *gapdh* as used as a standard. The proportion of PBMC which carry the provirus was estimated as follows: (copies of *tax)*/(2*copy number of gapdh)*100. Where > 1 copy of Tax is detected per 2 copies of GAPDH, the value exceeds 100%. Linker-mediated (LM)-PCR, high-throughput sequencing, data extraction and analysis of viral integration sites were carried out as described in Gillet et al[12]. Random fragments of genomic DNA (1 μg) generated by sonication were ligated to a partially double-stranded DNA adaptor. Nested PCR (two rounds) was used to amplify the region between the HTLV-1 LTR and the adaptor. Amplicons generated from adaptors with unique 6bp barcodes were combined into libraries; following which, sequence data from paired-end 50 bp reads and a 6 bp index (barcode) read were acquired on an Illumina HiSeq/MiSeq platform. Paired reads were then aligned to a human genome reference (Hg18). The number of individual cells which were sequenced within a given HTLV-1 infected clone were estimated by quantifying the number of distinct genomic shear sites generated by sonication (read2) for each paired unique integration site (junction between the provirus and human genome- read 1), and correcting to a calibration curve. The absolute abundance of unique integration sites per 100 PBMC was estimated by combining the proviral load and relative abundance of each clone.

The oligoclonality index (OCI) was used as a metric to compare the clone frequency distribution between samples. This was based directly on the Gini index [51], which calculates the relative inequality within a given distribution. The OCI was computed using the reldist package (http://CRAN.R-project.org/package=reldist) in R (http://www.R-project.org/). Values range between 0 and 1, with 0 indicating that all clones make up an equal proportion of the load, and 1 indicating that a single clone dominates completely [12].

### Flow cytometric analysis

Flow cytometric staining was performed as previously described [50] using panels of antibodies and stains outlined in S2 Table. Cells (3x 10^5^-2x10^6^) were stained for 5 min with 1 μl/ml fixable Live/Dead blue viability stain (Life technologies). After incubation cells were washed once with FACS buffer (PBS containing 7% normal goat serum). Surface molecules were stained for 20 min at room temperature (RT) with the antibodies listed in S2 Table. In order to quantify the frequency of T cells utilising each TCRVβ subunit, eight PBMC samples were stained with three anti-TCRVβ antibodies in parallel using the Beckman Coulter IOTest Beta mark kit. For the CD8^+^ killing experiments, PBMC were stained with an anti-TCRVβ antibody specific for the subunit most frequently utilised in that donor. Biotinylated antibodies were detected by staining with streptavidin-PeCy7 or -BV421 (Biolegend), 10 min at RT in FACS buffer. To stain intracellular antigens, cells were fixed and permeabilised using FoxP3 staining buffers (eBioscience, SanDiego), and stained with anti-Tax AF488 or anti-FoxP3 for 25 min at RT. Cells which were surface stained only were fixed with 2% paraformaldehyde in PBS for 20 min at RT. Data was acquired using a BD LSRFortessa, and analysed using Kaluza software. Gating strategy is outlined in S7 and S8 Figs.

### Analysis of TCRVβ oligoclonality

The frequency of live CADM1^+^CD4^+^CD3^+^ cells which bound each anti-TCRVβ antibody was expressed as a percentage of total live CD3^+^ T cells. In order to estimate the frequency of T cells expressing Vβ subunits which were not recognised by antibodies in the panel, the sum of all positively identified TCRVβ subunits was subtracted from the total frequency of live CADM1^+^CD4^+^ within CD3^+^ cells. The frequencies of TCRVβ-expressing live CADM1^+^CD8^+^CD3^+^ cells were also calculated in the same manner, as CADM1^+^CD8^+^ cells are also heavily infected with HTLV-1 [36]. The resulting 50 frequencies (including instances where a particular population was undetectable within CD3^+^ cells), were used to compute the oligoclonality index as described for the proviral integration site data. To avoid introducing sampling error in the case of low PVL (and thus low frequencies of CADM1^+^ cells) flow cytometric data from donors for which <500 CADM1^+^ events were acquired were excluded from this analysis.

### Cell survival assay

CD8^+^ cells were isolated from cryopreserved PBMC by positive selection using magnetic beads (Miltenyi Biotech) following the manufacturer’s protocol. The CD8^+^ fraction was placed in culture at 5 x10^5^ cells/ml for 13 days in the presence of 1 μg/ml phytohemagluttinin-L (Sigma Aldrich) and 100 IU/ml IL-2 (Promocell). At three day intervals, 50% of the culture medium was replaced and supplemented with 100 IU/ml IL-2. Cells were split as required. Flow cytometric analysis indicated that the mean frequency of live CD8^+^ cells in each culture was 99.6%; with a mean residual contamination of on average 0.28% of the ATL clone from that donor. On day 13, CD8^+^ cells were depleted from a second vial of cryopreserved PBMC. Cultured or freshly isolated ex-vivo CD8^+^ cells were added to between 3x10^5^ and 5x10^5^ CD8^−^ PBMCs at a range of effector: target (E:T) ratios in duplicate: CD8-depleted), the natural CD8^+^:CD4^+^ ratio (median 1:23), 1:4 and 1:2 as permitted by the number of CD8^+^ cells recovered. As significantly greater numbers of cultured CD8^+^ cells were recovered (versus ex vivo CD8^+^ cells) ratios of 1:1, 2:1 and 4:1 were tested where possible. Cells were co-cultured 1ml RPMI containing 10% FCS, 2 mM L-glutamine, 50 U/ml penicillin, 50 μg/ml streptomycin (Gibco) and 20 μg/ml DNAse (Sigma). After 18h, a 100 μl sample of each culture was harvested in order to count the absolute numbers of CD3^+^, CD4^+^ and CD8^+^ cells present. This was performed by adding 50 μl of an antibody master mix containing 1 μl anti CD8 AF700, 0.5 μl anti-CD3 BV510 and 0.25 μl anti-CD4 BV605 to each sample. Samples were incubated at RT for 30 min, after which 150 μl 2% paraformaldehyde in PBS was added, without any centrifugation/washing steps. Prior to flow cytometric analysis, 10 μl of CountBright absolute counting beads (Life Technologies) were added to each tube. The number of cells surviving was calculated as follows: # cells in tube = (# cells collected / # beads collected) × total # beads added to the tube. The remaining portion of each sample was analysed by flow cytometry as described above using the panel of antibodies outlined in S2 table.

### Analysis of cell survival assay

The relative frequency of cells in each subset was obtained using the gating strategy outlined in S8 Fig. If the total number of CD4^+^ cells in the tube changed during the course of experiment, frequencies were normalised to the absolute count of CD4^+^ cells in the CD8-depleted culture condition. In addition, the exact E:T ratio (total CD3^+^CD8^+^ cells: total CD3^+^CD4^+^) which was achieved in the co-culture was quantified in each case.

### Estimation of the rate of lysis of target populations

The rate at which cells in a given subpopulation of cells were cleared (% target cells killed/%CD8/day) was estimated in each subject as described in Asquith et al [46] using the following equation: *dy/dt* = *c- εyz*; where y is the percentage of targets within total CD4^+^ cells, c is the rate of antigen presentation (assumed to be constant during the short-term culture), ε is the CD8^+^ cell-mediated lytic efficiency, and z is the proportion of CD3^+^ cells that are CD8^+^. This model was solved analytically and fitted to the data using nonlinear least-squares regression (SPSS v22).

## Supporting Information

S1 Table Blood donor information. (XLSX)

S2 Table Antibodies used. (XLSX)

S1 Fig Frequency of TCRVβ subsets. (A) Frequency of CD4^+^TCRVβ^+^ populations as a percentage of total live CD3^+^ cells. (B) Size of largest possible clone by ATL subtype.
(TIF)

S2 Fig Normal range of maximum clone frequency in non-malignant HTLV-1 infected donors. (TIF)

S3 Fig High frequencies of CCR4^+^CADM1^+^CD7^−^ cells in donors with ATL. (TIF)

S4 Fig Example staining for HLA-ABC, Tax, FoxP3 and PD-L1. (TIF)

S5 Fig PD-L1 and FoxP3 expression. (TIF)

S6 Fig Killing of ATL cells and non-malignant infected cells is inhibited in the presence of 20 nM Concanamycin A. (TIF)

S7 Fig Gating strategy for TCRVβ flow cytometric analysis. (TIF)

S8 Fig Gating strategy used in cell survival assay. (A) Gating strategy used for multiparameter analysis (B) Gating strategy for absolute T cell counts.
(TIF)
