# Supplementary figures and images for: T Cell Receptor Vβ Staining Identifies the Malignant Clone in Adult T cell Leukemia and Reveals Killing of Leukemia Cells by Autologous CD8+ T cells
Source: PLoS Pathog. 2016 Nov 28;12(11):e1006030. doi: 10.1371/journal.ppat.1006030 (PMC5125714; doi:10.1371/journal.ppat.1006030)

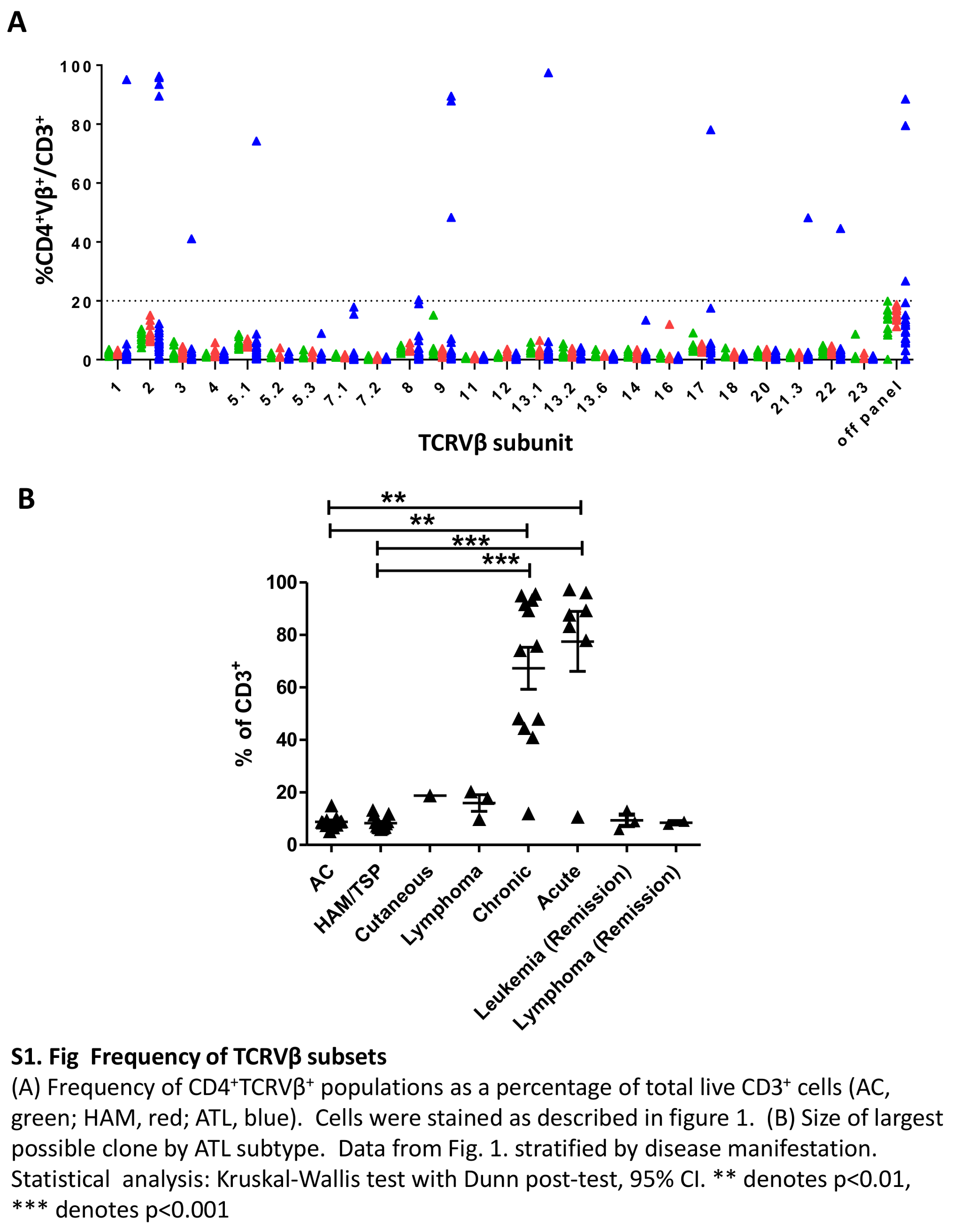

Supplement: S1 Fig — (A) Frequency of CD4+TCRVβ+ populations as a percentage of total live CD3+ cells. (B) Size of largest possible clone by ATL subtype. (TIF) [file ppat.1006030.s003.tif]

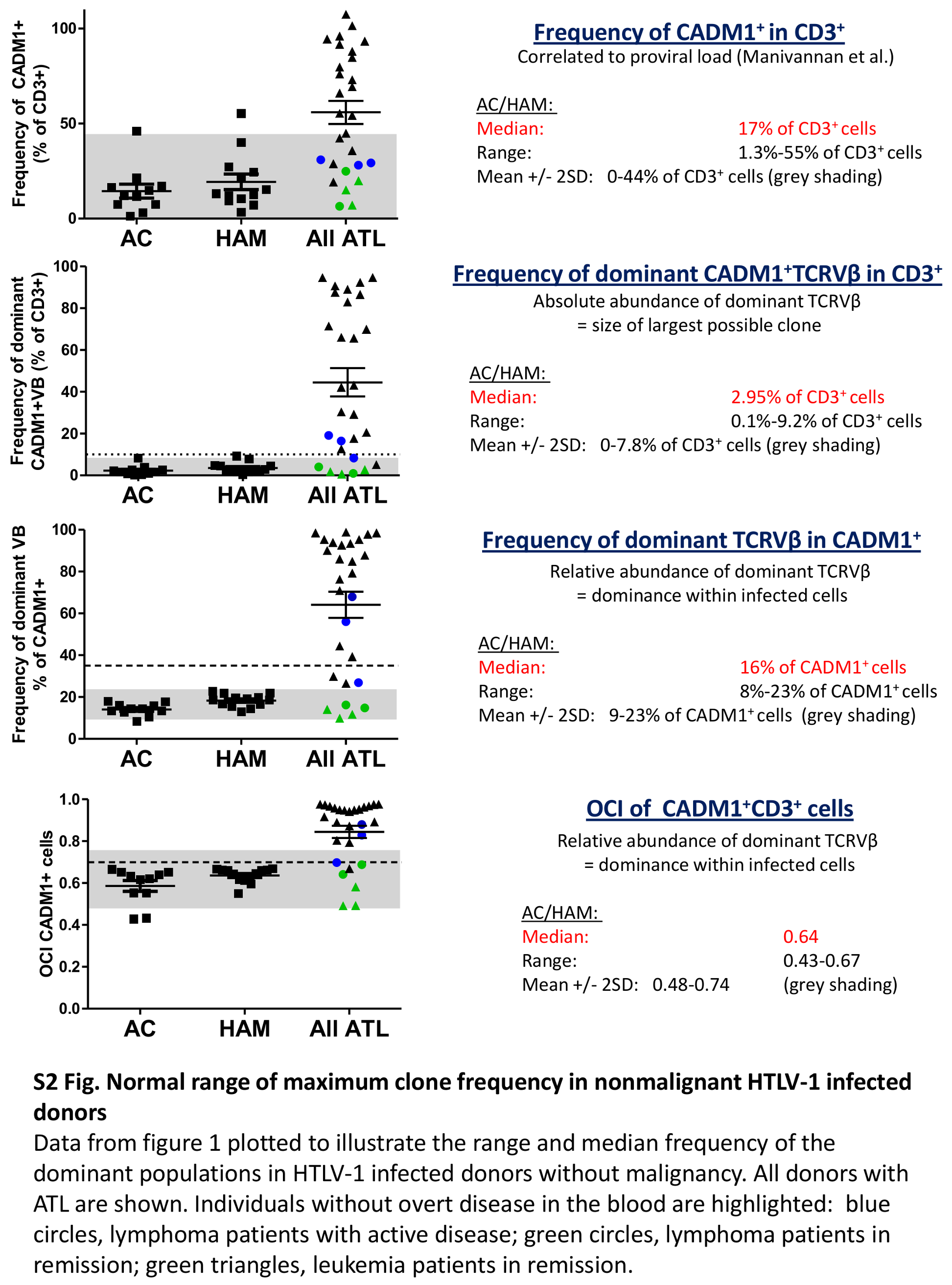

Supplement: S2 Fig — (TIF) [file ppat.1006030.s004.tif]

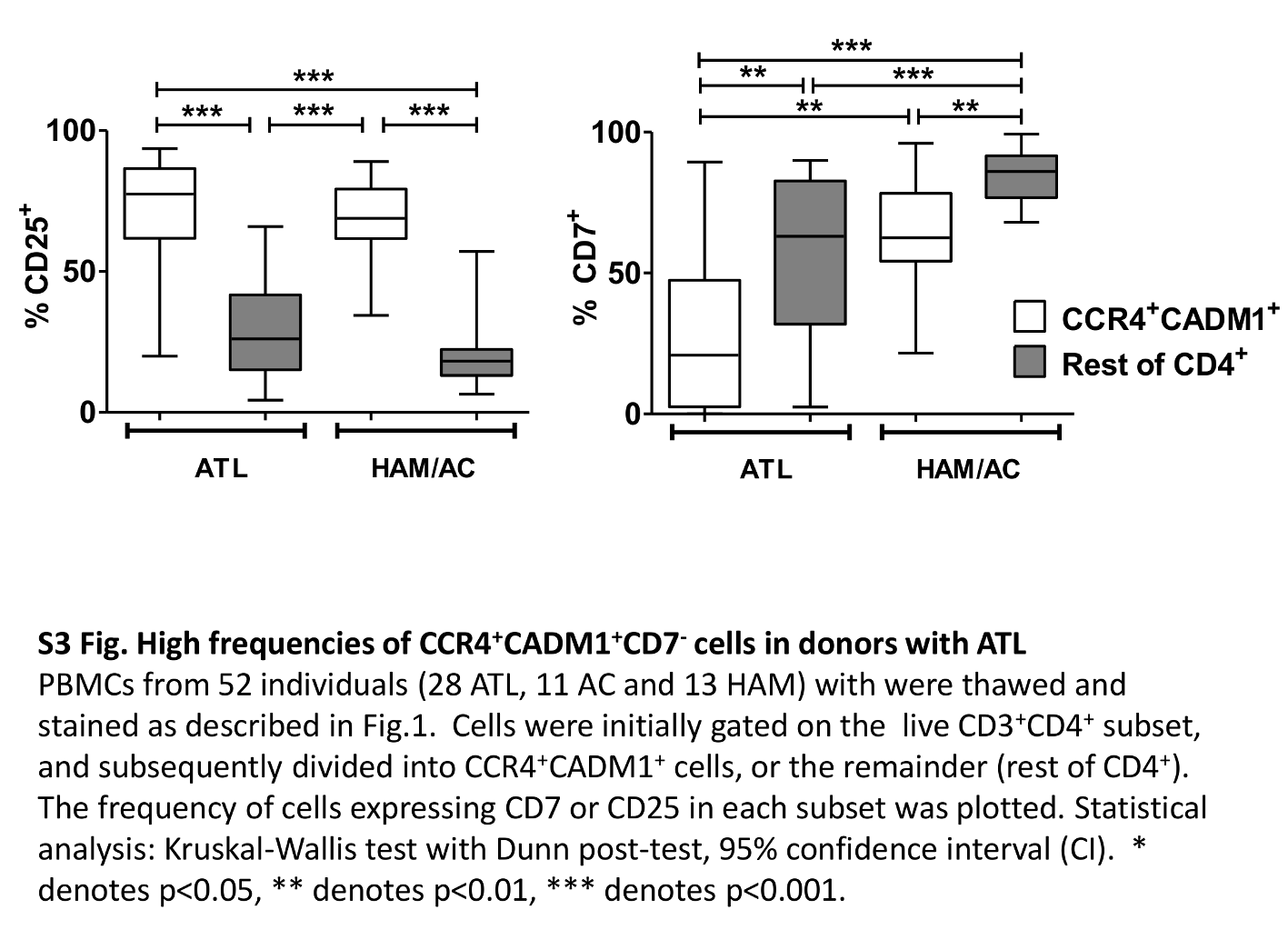

Supplement: S3 Fig — (TIF) [file ppat.1006030.s005.tif]

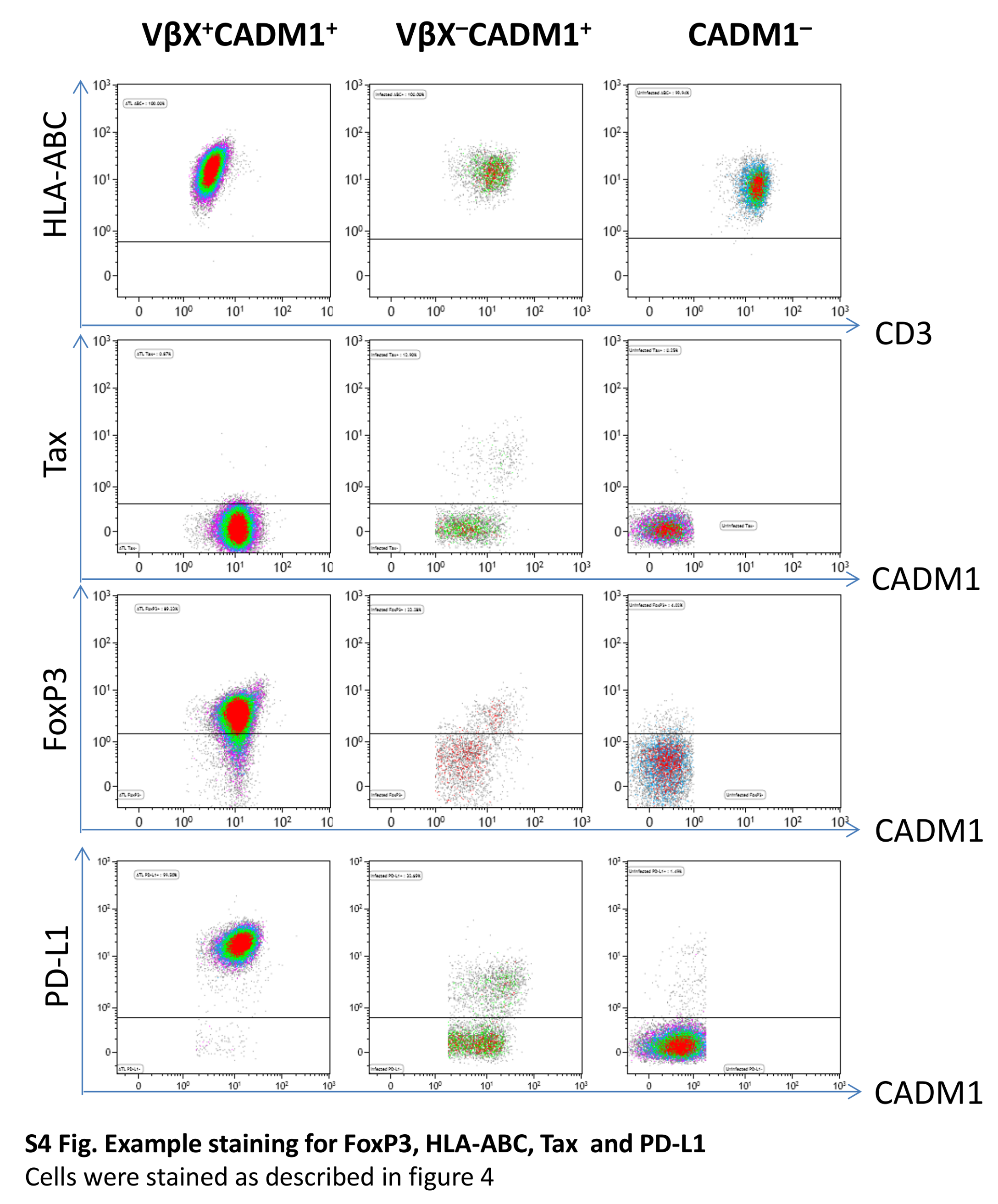

Supplement: S4 Fig — (TIF) [file ppat.1006030.s006.tif]

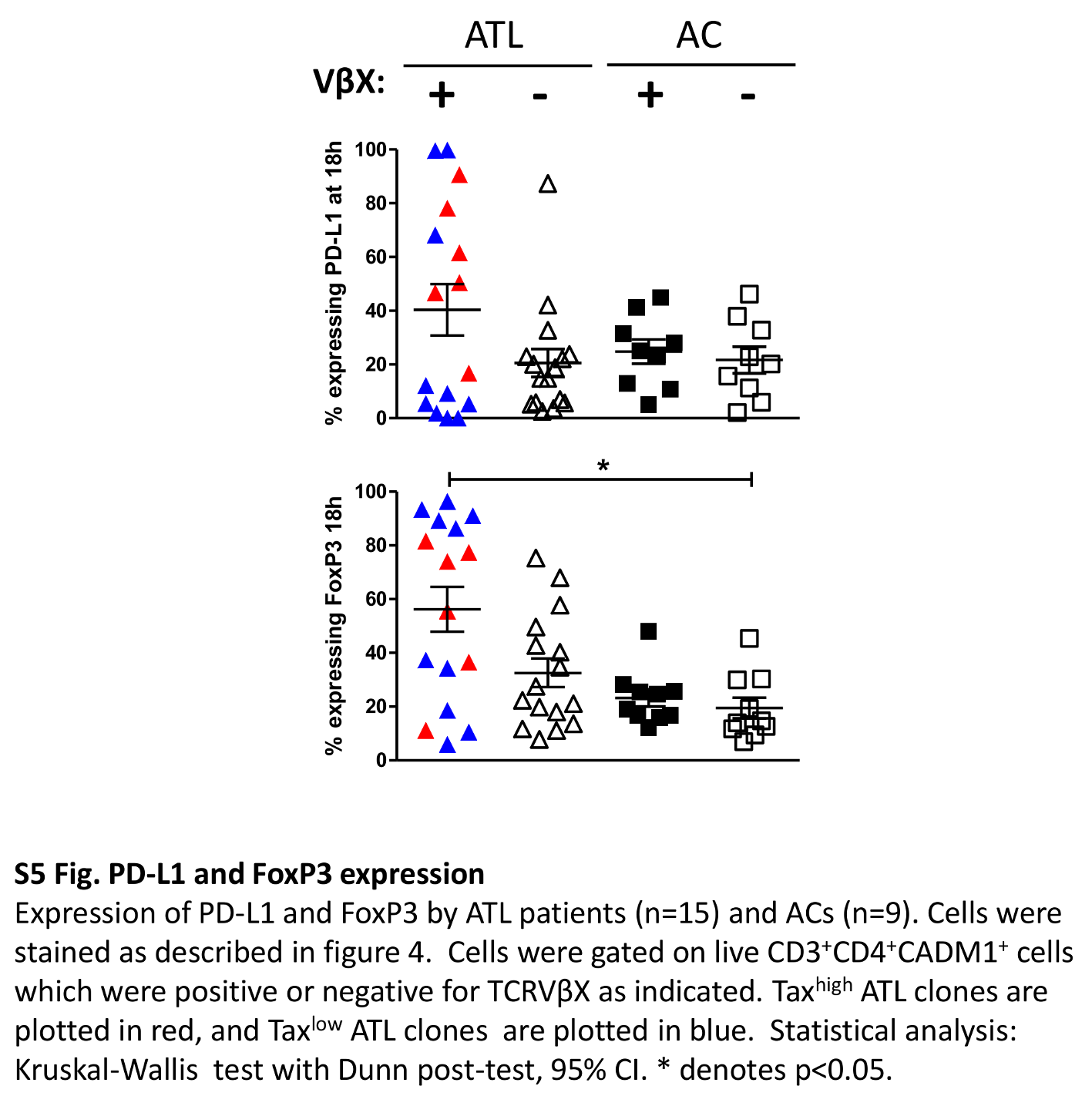

Supplement: S5 Fig — (TIF) [file ppat.1006030.s007.tif]

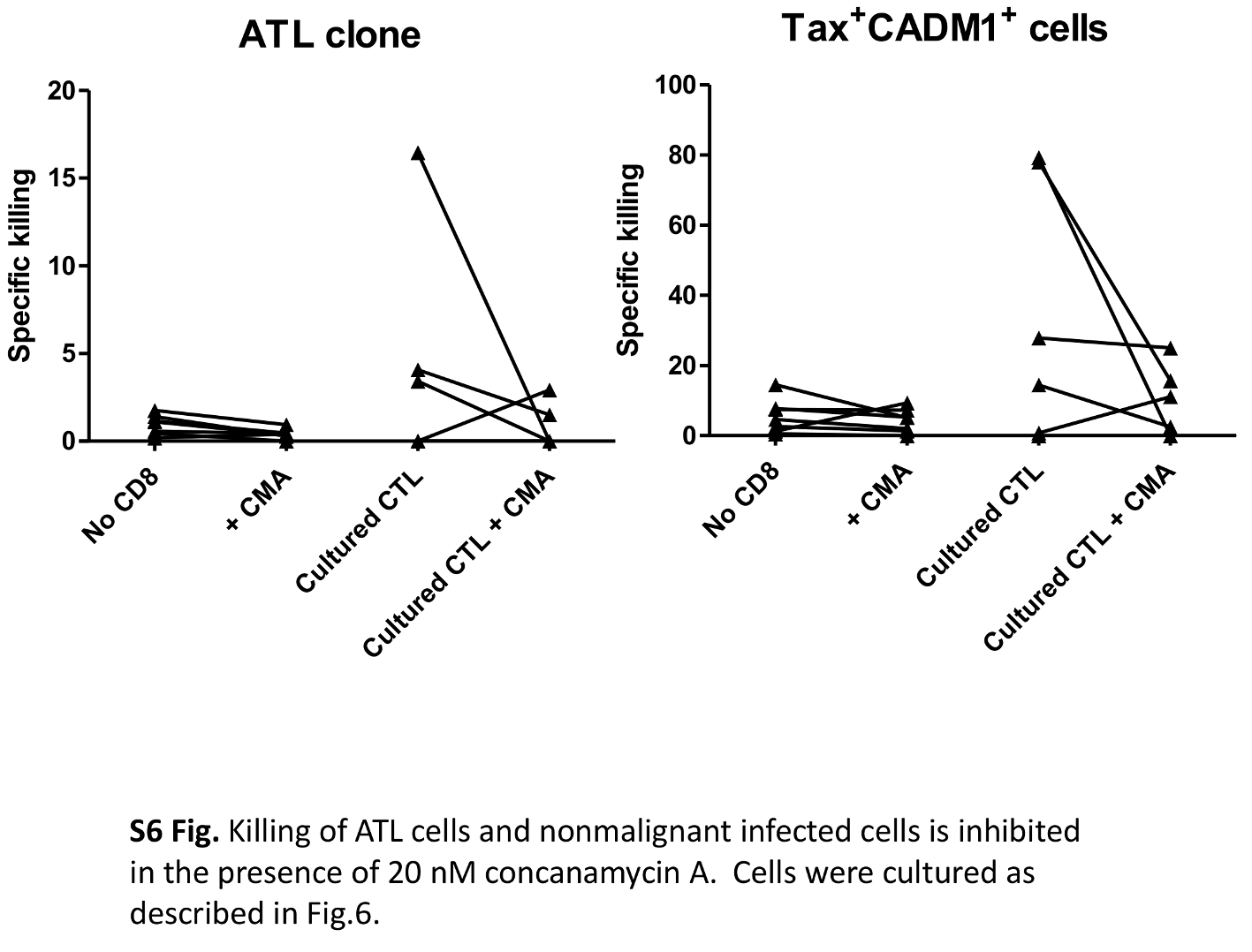

Supplement: S6 Fig — (TIF) [file ppat.1006030.s008.tif]

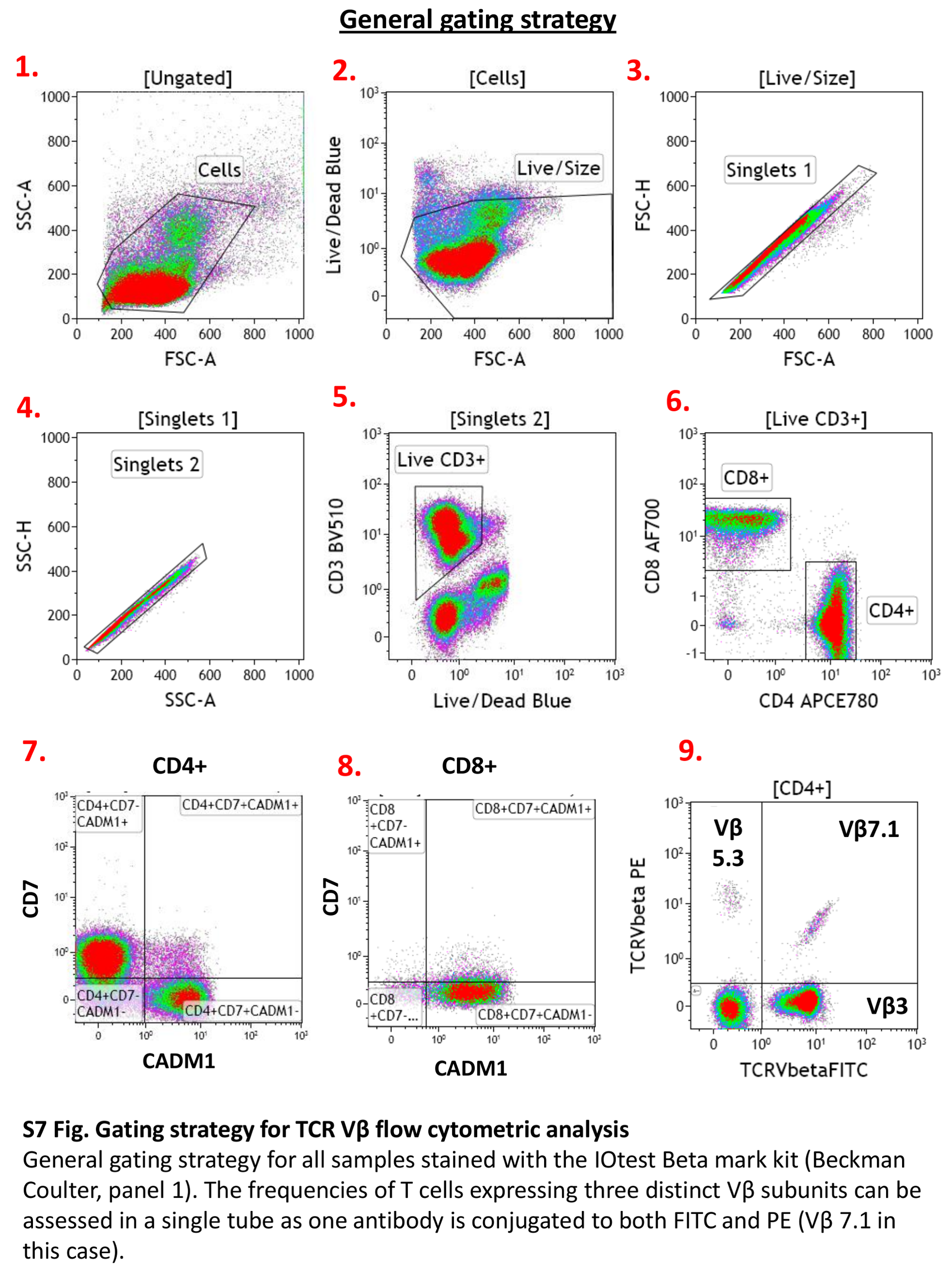

Supplement: S7 Fig — (TIF) [file ppat.1006030.s009.tif]

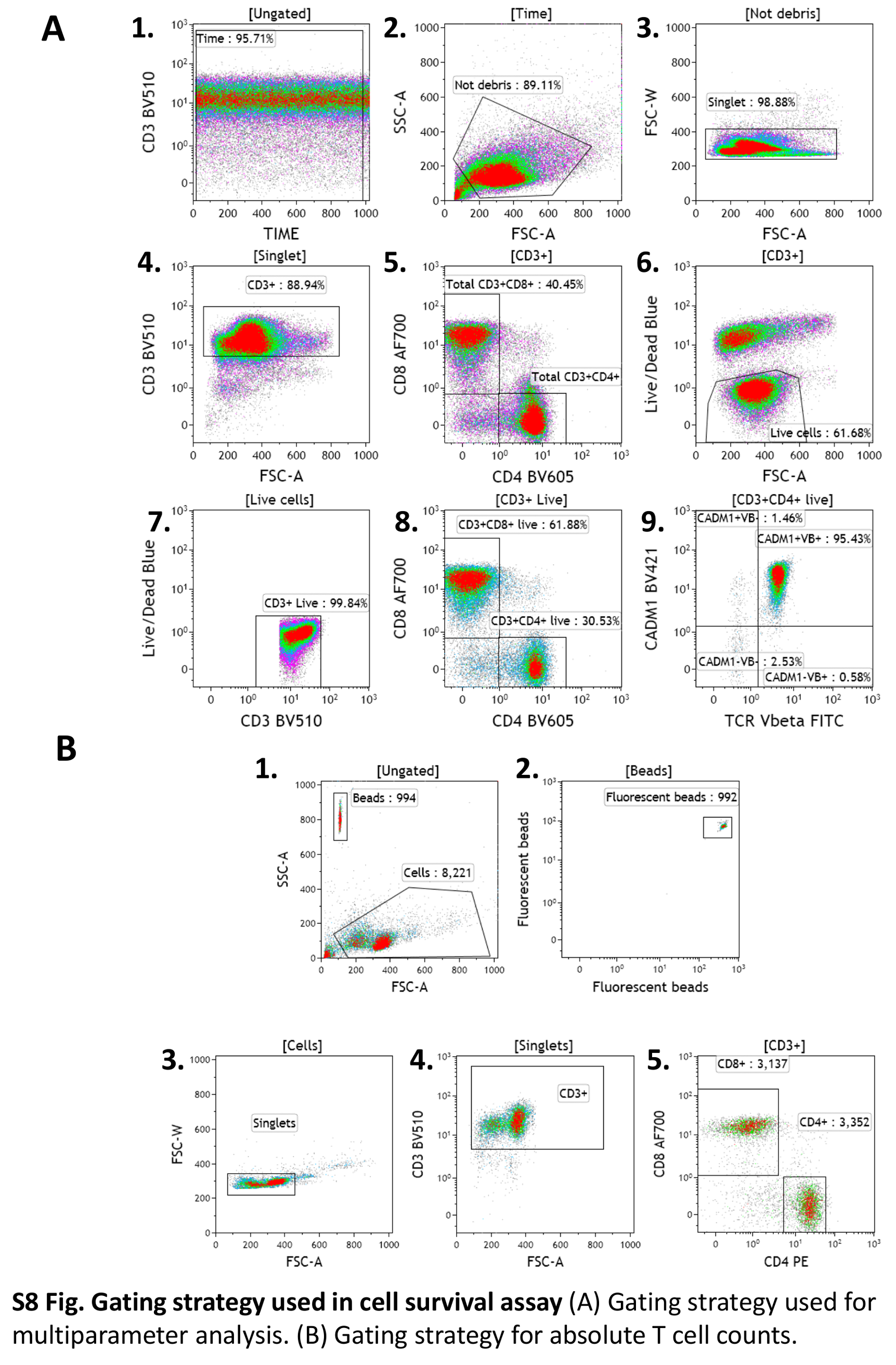

Supplement: S8 Fig — (A) Gating strategy used for multiparameter analysis (B) Gating strategy for absolute T cell counts. (TIF) [file ppat.1006030.s010.tif]
